# Professionally designed information materials and telephone reminders improved consent response rates: evidence from an RCT nested within a cohort study

**DOI:** 10.1016/j.jclinepi.2015.03.014

**Published:** 2015-08

**Authors:** Andy Boyd, Kate Tilling, Rosie Cornish, Amy Davies, Kerry Humphries, John Macleod

**Affiliations:** ALSPAC, School of Social and Community Medicine, University of Bristol, Oakfield House, Oakfield Grove, Bristol BS8 2BN, UK

**Keywords:** Recruitment, Retention, Randomized controlled trial, Cohort study, Record linkage, ALSPAC

## Abstract

**Objectives:**

To investigate whether different study-to-participant communication methods increase response, increase response from hard-to-engage individuals, and influence participants' consent decisions.

**Study Design and Setting:**

A randomized controlled trial within the Avon Longitudinal Study of Parents and Children. Cohort members were invited to re-enroll at age 18 and consent to linkage to their health and administrative records. Participants were randomized to receive one of eight combinations of three interventions: a prior-notification postcard or no contact, a standard or professionally designed consent pack, and a phone or postal reminder. The primary outcome was return of the consent form (“response”), with consent decision being the secondary outcome.

**Results:**

Of 1,950 participants, 806 (41%) responded. Response rates were 2.7% higher (95% confidence interval: −0.06, 5.5%; *P* = 0.06) among those receiving designed packs than among those receiving standard packs and 6.4% higher (2.3, 10.6%; *P* = 0.002) among those receiving phone reminders (compared with postal reminders). The prior-notification postcard did not influence response rates [difference = 0% (−2.8, 2.8%; *P* = 1.0)], and we found no evidence that the communication method influenced consent decision.

**Conclusion:**

This trial provides evidence that communication material design can influence response rates and that phone reminders have superior cost/benefit returns over designed materials. Experimental evaluation of communications strategies and dissemination of findings may benefit cohort studies.

## 1 Introduction

**What is new?**

• Professionally designed information materials and phone reminders increased response rates, whereas prior-notification mailings did not alter the rate of response.
• We found no evidence that the participant contact method changed the nature of the response (i.e., whether consent was granted or withheld).
• We recommend that studies should implement professionally designed information packs and conduct phone reminders during recruitment and consent campaigns, but studies should prioritize phone reminders where funding is limited.
• Subgroup analyses suggested that phone reminders may improve response rates from individuals with low educational attainment, a group known to be more susceptible to attrition from cohort studies.
• This article demonstrates the value for cohort studies of routinely testing participation strategies using rigorous methods to improve their effectiveness and the exchange of information between studies.

High levels of participant recruitment and retention are critical to the success of any cohort study [1], [2]. Problematic recruitment or retention can introduce bias into study findings and uncertainty as to how to interpret results in a clinical context [3]. Recruitment and retention rates vary across studies and are differentiated by health and socioeconomic status and demographic characteristics [4], [5], [6], [7], [8], [9], [10]. Response to requests for consent to using health records is differentiated by similar patterns [11]. To encourage recruitment and retention, studies enter into a dialog and long-term relationship with participants. Studies provide information about their aims, methods, and findings and, in turn, seek to collect data and consent to use participants' personal information. Yet, the study information materials used to do this, and the process by which they are implemented may hinder recruitment or ongoing participation if implemented poorly.

Material design can be improved through public and participant consultation, engaging professional expertise and considering response behavior theories [12]. Experimenting with consent methodologies can be justified both from a participant perspective (improving informed consent) [13] and from a scientific perspective (in terms of improving sample size and reducing possible selection bias) [14]. Existing evaluations, in differing research settings, have found a wide range of strategies that modify recruitment/response in population studies: (1) monetary incentives, questionnaire content, design and length, personalized communication, and mode of questionnaire delivery (e.g., recorded delivery, envelope design) increased questionnaire response [15]; (2) increasing awareness of the health conditions under investigation, possible implications for participant health, and engaging individuals in the trial process may increase recruitment to randomized controlled trials (RCTs) [16]; (3) the provision and clarity of study information is important to encourage enrollment into RCTs [17]; and (4) offering incentives boosted retention rates in cohort studies [18]. Studies seeking to improve response from clinicians have produced similar evidence [17], [19], [20], [21]. However, options to use such strategies are limited by ethicolegal requirements when used in recruitment or requests for consent.

Most evidence to date considers adult participation in RCTs rather than cohort studies, meaning there is a lack of rigorous evidence regarding strategies to retain participants and increase response rates in cohort studies [18], where recruitment and retention issues may differ. This can be addressed using RCTs to compare different strategies. However, studies may be reluctant to experiment due to limited resources and concerns that varying strategies may influence the nature of the response (e.g., the consent outcome or data collection value) rather than just the response rate [18]. Through the Project to Enhance ALSPAC through Record Linkage (PEARL), we sought to re-enroll the index children of the Avon Longitudinal Study of Parents and Children (ALSPAC) into ALSPAC as they reached adulthood. We also sought consent to amend ALSPAC to include the use of data from participants' routine health and administrative records as a new means of data collection. We compared three variations of how we communicate this consent request using an RCT methodology. Specifically, the trial tested three hypotheses: whether study information material design and format: (1) affect the absolute response rate, (2) differentially affect response rates from those participants with demographic characteristics predictive of nonresponse, and (3) impact on the nature of the response (whether consent is given). There is a need to embed such RCTs within ongoing cohort studies at different ages to inform future study design and maximize the value of cohort studies.

## 2 Methods

### 2.1 Avon Longitudinal Study of Parents and Children

ALSPAC is a transgenerational prospective birth cohort study. ALSPAC recruited pregnant women resident in and around the City of Bristol (South-West UK) and due to deliver between April 1, 1991, and December 31, 1992. By the time the index children had reached age 18, ALSPAC had recruited the mothers of 15,247 pregnancies, which resulted in 15,458 fetuses. Of these, 14,775 were live births and 14,701 were alive at 1 year of age. The cohort has been followed intensively from birth through self-completed questionnaires and attending clinical assessment visits. ALSPAC has built a rich resource of phenotypic and genetic information relating to multiple genetic, epigenetic, biological, psychological, social, and other environmental exposures and outcomes (the ALSPAC Web site hosts a data dictionary that describes the available data) [22]. ALSPAC's eligibility criteria and recruitment rates are described in depth elsewhere [4]. Study participation varies from single contributions by any family member to complete contributions from all family members and from those whose first involvement occurred recently to those who have not participated for many years [4]. Historically, ALSPAC index children provided assent to data collection from age 9, although parental consent was mandatory until age 16. Because of conflicting ethics committee decisions regarding the appropriate age to formally re-enroll the children into ALSPAC, it was decided to seek re-enrollment once the child reached legal adulthood (age 18). Enrollment is defined as providing ALSPAC with permission to maintain an administrative record of the participant on the study database and contact the participant to invite them to participate in future data collection; participants are not asked to commit to providing any data.

### 2.2 Project to Enhance ALSPAC through Record Linkage

PEARL is a Wellcome Trust funded study (running between 2009 and 2016) that aims to develop generalizable methods for cohort studies to use routine records using data linkage techniques. PEARL uses ALSPAC as an exemplar setting for this research and therefore has the secondary aim of enhancing ALSPAC through linking health and administrative records (education, benefits and earnings, and criminal convictions and cautions records) to the ALSPAC databank. PEARL sought to inform participants of its aims and to collect evidence of appropriate participant permissions. This was scheduled to run after the index children reached adulthood. It was decided to combine the request for the index children to re-enroll into ALSPAC and the request for ALSPAC (via PEARL) to use participants' routine records into one postal consent campaign. All index children from families enrolled into ALSPAC, except those who had died, withdrawn from the study once 14 years of age or could not be traced are considered eligible for PEARL. Thus, the PEARL catchment area included the whole of the UK.

### 2.3 RCT of PEARL consent materials

**Fig. 1:**
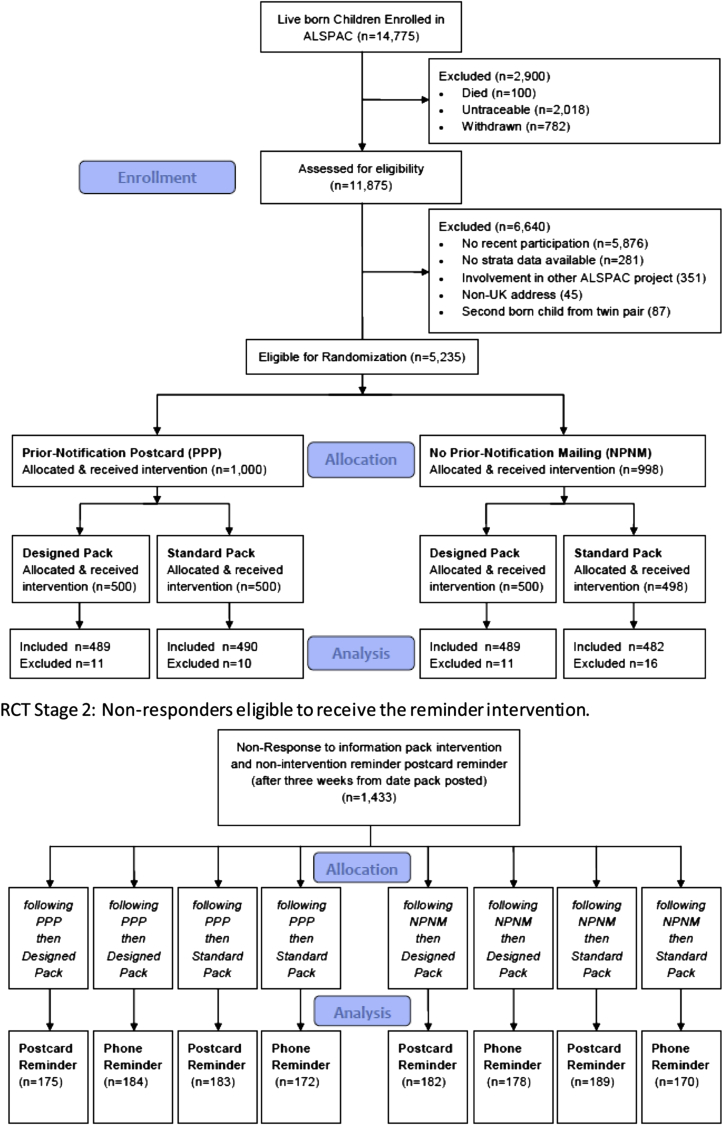
RCT sample selection and allocation to intervention groups. RCT, randomized controlled trial; ALSPAC, Avon Longitudinal Study of Parents and Children.

The RCT evaluated three variations in how consent materials were provided. These were sent to the PEARL RCT sample, which was drawn from the full PEARL sample (Fig. 1). As differing participation histories could complicate the interpretation of our results, we restricted the PEARL RCT sample selection to young adults who had recently participated (those attending ALSPAC clinical assessments aged 15 or 17 or returning the 16-year questionnaire). To control for differing extents of individual participation history, we stratified our sample using a participation score (see Section 2.4). Where an individual selected for the trial was a twin, we allocated the nontrial twin to the same intervention groups and administered the interventions as if they were part of the trial. Nontrial twins were excluded from analysis. The RCT was conducted from April 18, 2011, until December 23, 2012.

The RCT primary outcome was the absolute response rate (i.e., whether a participant returned the consent form), and the secondary outcomes were: (1) re-enrollment rates to the study and (2) consent rates for ALSPAC's request to collect data from routine records (described in depth in 2.7). Information materials were sent to participants describing the study and data collection from routine records. Participants were asked to consider the information, ask questions if required (ALSPAC fieldworker phone, mobile phone, SMS text message, and email addresses were provided), and to return a single consent form (which asked multiple, separate, consent questions) using a prepaid envelope. To aid clarity [23], we split the information materials into four “layers” of detail: a covering letter, a four-page summary leaflet, a 32-page detailed booklet supported by detailed Web pages. Materials were tested for clarity using Flesch-Kincaid readability metrics [24] and by participant focus groups. To meet concerns regarding literacy levels, an audio version of the materials was included in the pack on a CD and made available via the Web site. These materials constitute the information “pack” (available from the ALSPAC Web site [25]). Most pack contents were ethicolegal requirements, and therefore, we did not alter the wording between the intervention groups; however, there was scope to alter the design of the materials and the means of reminder follow-up.

### 2.4 Ethics

Ethical approval for ALSPAC was obtained from the ALSPAC Ethics and Law Committee and the Local Research Ethics Committees. PEARL, including this trial, received approval from the Haydock NHS Research Ethics Committee. Explicit participant consent for trial involvement was not sought (nor required by the ethics committee); however, we notified participants about the RCT and trial aims in the study information materials.

### 2.5 Interventions

**Fig. 2:**
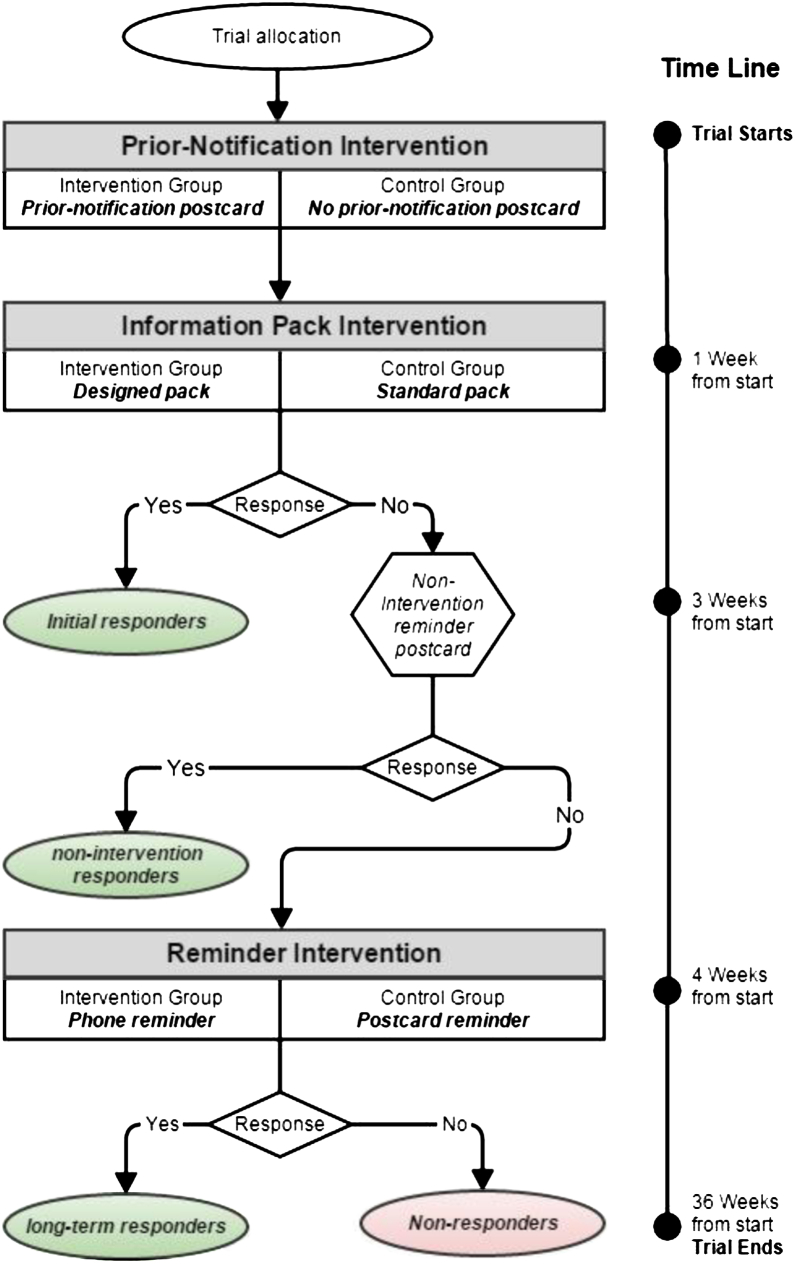
Summary of the PEARL RCT interventions. PEARL, Project to Enhance ALSPAC through Record Linkage; RCT, randomized controlled trial.

**Fig. 3:**
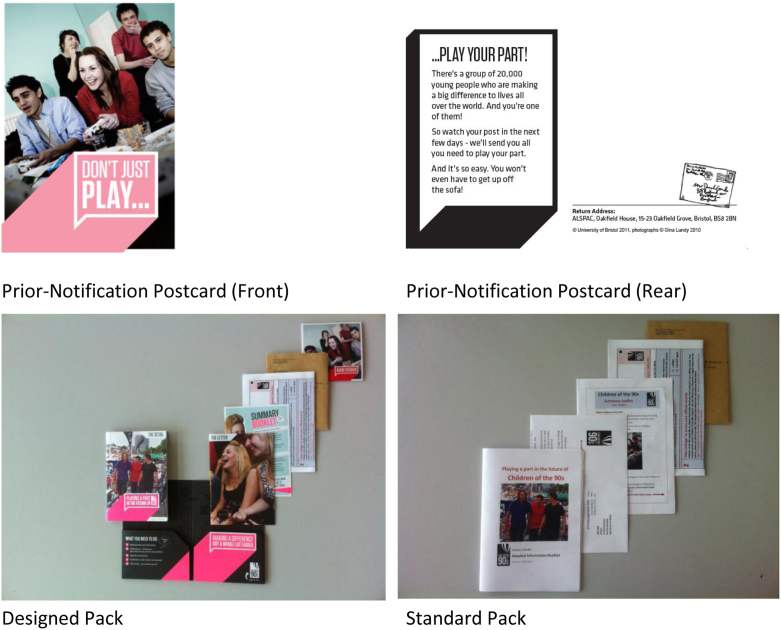
Prior-notification postcard and information pack intervention designs.

Through PEARL, we developed three interventions (the intervention processes and naming conventions are summarized in Fig. 2), each based on a comparison between ALSPAC's standard approach and a modified approach thought likely to improve response to the re-enrollment and consent request or to encourage response from the harder-to-engage groups. The first intervention (hereafter the “prior-notification intervention”) consisted of a postcard (hereafter “prior-notification postcard”), sent to individuals in the intervention arm a week before the second intervention (Fig. 3). The comparison group received no prior contact (hereafter “no prior-notification mailing”).

The second intervention (hereafter “information pack intervention”) compared a professionally designed information pack (hereafter “designed pack”) against a standard version (hereafter “standard pack”) (Fig. 3). The designed pack comprised a folder, which contained a cover letter, summary and detailed booklet, audio CD, consent form, and prepaid envelope. The standard pack included the same elements (excluding the folder) but was produced by the authors using Microsoft Word (Redmond, WA, USA). Although it contained the same text and principal photographs as the designed pack, its appearance was plain in comparison. Two weeks after the packs were sent, a reminder postcard was mailed to all nonresponders. This mailing was not a tested intervention (hereafter: “nonintervention reminder postcard”).

Three weeks after the information pack intervention, nonresponders were eligible to receive the third intervention (hereafter the “reminder intervention”). Individuals in the intervention arm received a reminder via phone, SMS text message, or email (hereafter simplified to “phone reminder”). The comparison group was sent a postcard reminder (hereafter “postcard reminder”). In the intervention arm, two attempts were made by ALSPAC fieldworkers to phone the participant, using mobile and home contact numbers, with one being made between 12 am and 5 pm and the other between 5 pm and 7 pm. If no contact was made, the participant was sent a text message to their mobile phone or, where no mobile number was held, they were sent a reminder email. If the young person's parent answered the phone, the fieldworker asked them to remind their child to return the mailing (messages were not left with any other family member). The fieldwork team conducting the phone reminder calls was trained in the methods and objectives of ALPSAC as well as in data linkage as a means of data collection. The phone reminder calls were not scripted; instead, fieldworkers were asked to make three key statements: (1) to thank the participants for their help and to say how valuable individual contributions were, (2) to ask whether there were any questions or concerns, and (3) to remind the participant to return the consent form. We recorded the call outcome, recipient, and any pertinent feedback or comments (e.g., requests for additional information or questions about the process).

### 2.6 Randomization and sample allocation

Randomization was stratified by sex, socioeconomic characteristics, and level of participation history. We used the English Indices of Multiple Deprivation 2007 (IMD) [26] as the indicator of socioeconomic position. We allocated the neighborhood IMD score into tertiles at a national level and then linked the tertile value to the participant using residential address. Using study records, we created a score characterizing the participant's historical level of study involvement. This “participation” score (*S*) was derived from attendance at study assessment clinics (*a*) and questionnaire response (*b*), in relation to the total number of clinics (*C*) and questionnaires (*Q*), where:

$$S=50\left(\frac{a}{C}\right)+50\left(\frac{b}{Q}\right)$$

A score of ≥90 was considered to be “very high”; “lower” participation cases were selected evenly from scores between 1 and 89. The equal strata of “very high” participation and “lower” participation was chosen to allow the assessment of response differences between historically “very good” responders and those who have contributed less frequently.

The RCT sample (*n* = 1,998) was randomly selected from the PEARL sample (*n* = 5,235 after selected individuals had been excluded, as described in Section 2.3) within the 12 strata (formed by sex, a binary measure of ALSPAC study participation, and IMD tertile). Randomization into the eight trial arms was then performed within each of these 12 strata. The allocation of individuals to interventions was conducted between A.B. and K.T. A.B. coded the stratification characteristics for each individual and pseudonymized the data. K.T. used the pseudonymized data to create the stratification groups and then randomly allocated each subject within each strata into one of the eight intervention groups (using the random number generator within Stata). A.B. allocated each group to an intervention arm, although the allocation sequence was still concealed. At the point of allocation, the participants were identified using the pseudonym key and K.H. administered the distribution and collation of the prior-notification and the information pack interventions. K.H. later administered the reminder intervention.

### 2.7 Statistical analysis

#### 2.7.1 Primary outcome

During analyses, we considered the prior-notification intervention and information pack intervention separately from the reminder intervention, on the basis that we did not intend to “treat” individuals who responded before the reminder was due. Thus, the prior-notification and information pack interventions were analyzed on an intention-to-treat (ITT) basis, and the reminder intervention was analyzed on a modified ITT (mITT) basis, where the analysis included only those who did not respond to the pack after the first reminder and a total elapsed time of 3 weeks (Fig. 1). The analyses were conducted with knowledge of the intervention received. Our primary outcome was the absolute response rate. We calculated both “initial response” (where the consent form needed to be received by ALSPAC before the nonintervention reminder postcard mailing and within 3 weeks from the prior-notification intervention send date) and “long-term response” (defined as returning the consent form after the reminder intervention was administered and up to the end of the trial). Response after the nonintervention reminder postcard and before the reminder intervention is not counted as either “initial response” or “long-term response.” Initial response is the outcome for the primary ITT analysis, whereas long-term response is the outcome in the mITT analysis (and initial responders are excluded from this mITT analysis). We used chi-squared tests to compare response rates between the intervention groups. The primary analyses were repeated using logistic regression to adjust for factors known to be predictive of nonresponse in ALSPAC (the stratification variables and educational attainment). No adjustments to *P*-values were made for multiple testing.

#### 2.7.2 Secondary outcomes

Our secondary analysis measured whether the design and format of the three interventions impacted on consent rates (where the outcome was whether consent was given for (1) re-enrollment and (2) linkage, as measured at the end of the trial). We used Fisher's exact tests to compare consent rates between the intervention groups.

#### 2.7.3 Subgroup analyses

Using logistic regression (including interaction terms), we conducted subgroup analyses to assess whether the three interventions differentially affected response rates among participants with factors known from a previous study to be predictive of nonresponse within ALSPAC [4]. These factors were covariates from the ALSPAC data set (sex and participation history), neighborhood deprivation (using tertiles of IMD), and variables from individual-level linkage to the National Pupil Database (NPD) Key Stage 4 (KS4) data set. The KS4 data set records pupil census and assessment data for pupils in English schools at mean age 16 (the last compulsory educational attainment assessment in the UK). ALSPAC linked 11,008 (72.2%) enrolled children to a subset of KS4 comprised government maintained establishments. This sample, which excludes privately funded and specialist care establishments, has an 89.5% coverage of English pupils nationally and 84.3% in the ALSPAC region. The KS4 measures of interest were: (1) a binary indicator of attainment (achieving five or more or less than 5 A*–C graded assessments—in the UK, this is commonly interpreted as the minimum threshold an individual needs to obtain to progress into post-16 education), (2) household income [child either eligible, or not, for free school meals (FSM), indicating a combined family income of ≤£16,000 per annum], and (3) ethnicity (aggregated to white and nonwhite). We used the “metan” Stata command to examine risk differences by the subgroups defined by these factors.

We kept a record of all costs relating to the trial interventions and conducted a comparison to establish the cost, per individual, of adopting any successful intervention methods. The data were analyzed in Stata v12 (StataCorp. 2011. Stata Statistical Software: Release 12. College Station, TX: StataCorp LP).

### 2.8 Sample size calculation

The PEARL RCT aimed to inform an optimal information material design to use in ALSPAC. An a priori power calculation of 1,998 was selected to balance required statistical power for the RCT and to enable the optimal material designs to be sent to most of the PEARL sample. This sample size was sufficient in the initial mailings (the prior-notification and information pack interventions) to detect a 7% difference in response with 83% power at 5% significance level.

## 3 Results

PEARL prior-notification and information pack interventions were administered to 1,998 participants during April and May 2011. Where there was evidence that the mailing was not received (the mailing being returned “addressee not known” by the postal service or participants requesting replacement mailings), we excluded the individual from the analysis [*n* = 48 (2.4%)], resulting in an analyzable sample of 1,950. The prior-notification postcard was sent to 50.2% (979/1,950) and the designed pack to 50.2% (978/1,950) of the analyzable sample. Three weeks after the information pack was posted, nonresponding participants (73.5%; *n* = 1,433) were deemed eligible to progress into the reminder intervention (including both intervention and control arms). The phone reminder was administered to 49.1% (704/1,433) of the nonresponders and the postcard reminder to the remaining 50.9% (729/1,433).

There was a total response rate of 41.3% (806/1,950) at the end of the PEARL trial. Of these, 10.9% (213/1,950) were initial responders, 15.6% (304/1,950) responded after the nonintervention reminder postcard mailing, and 14.8% (289/1,950) were long-term responders, who responded after the reminder intervention (see Supplementary Figure 1, in Supplementary Materials at www.jclinepi.com).

### 3.1 Response patterns by intervention

There was no evidence that receiving the prior-notification intervention before the information pack intervention altered initial response rates compared with receiving no prior mailing (0.01% difference in response; 95% confidence interval (CI): −2.8%, 2.8%; *P* = 1.0; Table 1). There was some evidence that initial response rates were higher (2.7% difference; 95% CI: −0.06, 5.5%; *P* = 0.06) where individuals were sent the designed pack in comparison with the standard pack.

Response to the nonintervention reminder postcard was similar regardless of whether individuals had received the designed pack or standard pack, or the prior-notification postcard or no prior-notification mailing (see Supplementary Table 1 at www.jclinepi.com). After the nonintervention reminder postcard and by the point at which the reminder intervention was due, 1,433 participants had not responded.

Using a mITT approach, we examined long-term response to the reminder intervention. Of those randomized to receive the phone reminder, there was a 23% response rate (165/704), in comparison with a 17.0% response rate (124/729) for those sent the postcard reminder (6.4% difference; 95% CI: 2.3, 10.6; *P* = 0.002; Table 2). The phone reminder was most effective (when compared with the postcard reminder) where an ALSPAC fieldworker spoke directly with the index child (16.0% difference; 95% CI: 8.6, 23.3%; *P* < 0.001) and was still effective when we left a message with a parent (8.0% difference; 95% CI: 1.5, 14.5%; *P* = 0.01). We found no evidence that individuals not contacted in the phone reminder group were less likely to respond than those receiving the postcard reminder (−0.4% difference; 95% CI: −5.7, 4.9; *P* = 0.9).

We found no evidence (Table 2) for an interaction between the prior-notification intervention and the information pack intervention (*P* = 0.1) or between the prior-notification intervention and the reminder intervention (*P* = 0.4). However, there was evidence for an interaction between the information pack intervention and the reminder intervention, suggesting that the phone reminder had a greater impact when the participant received the standard pack (*P* = 0.05).

### 3.2 Subgroup analyses

The sample stratification, which oversampled participants with very high levels of participation, means the RCT sample differs from the ALSPAC sample in terms of sociodemographic composition (Supplementary Table 2 at www.jclinepi.com). The sociodemographic characteristics of all responders (i.e., those who responded at any stage throughout the trial) differed from all nonresponders in each of the measured categories. Differences are similar to those observed in recent ALSPAC data collections [4]—in summary, all respondents are more likely to be female, white, have higher educational attainment and less likely to come from low-income households (see Supplementary materials at www.jclinepi.com for all response rates by sociodemographic characteristics).

**Fig. 4:**
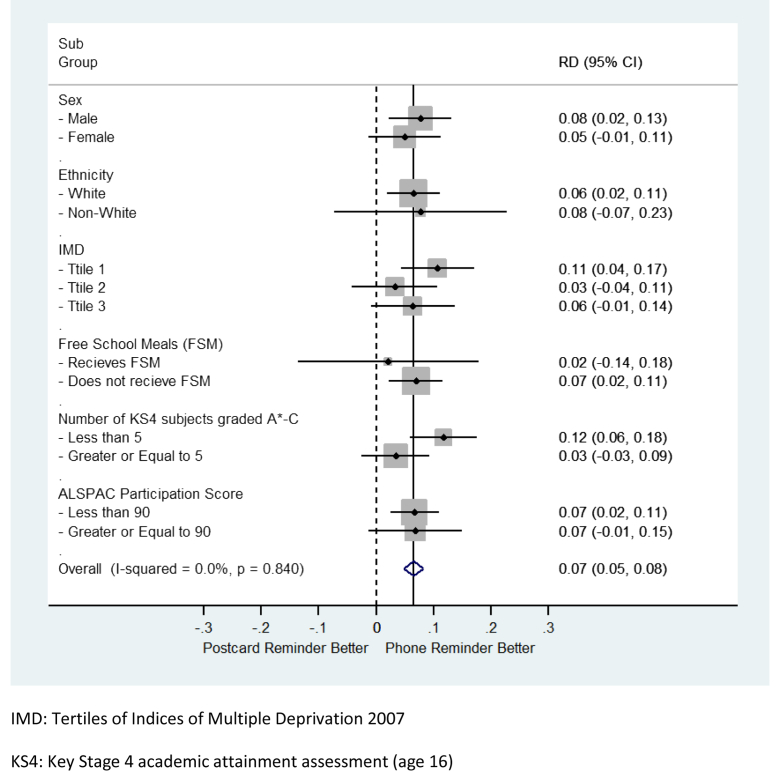
Subgroup analysis: differences in response for the reminder intervention by sociodemographic characteristics. CI, confidence interval; RD, risk difference.

We found no evidence to suggest that the effect of the prior-notification intervention or the information pack intervention differed according to any of the demographic and social factors shown previously to be associated with participation in ALSPAC (see Supplementary Table 3 at www.jclinepi.com). However, there was evidence that the phone reminder had more of an impact on response rates among individuals with lower educational attainment (Fig. 4). We observed an increase in long-term response from those with fewer than 5 A*–C grades of 11.7%, compared with a 3.5% increase in long-term response from those with five or more A*–C grades for phone vs. postcard reminder (*P*-value for interaction = 0.004). A similar pattern was observed when comparing the impact of the phone reminder among participants living in the most deprived neighborhoods to that among participants living in the least deprived neighborhoods (10.7% difference vs. 6.4% difference, respectively, *P*-value for interaction = 0.09). However, when we controlled for educational attainment, the interaction effect did not remain (*P* = 0.2), suggesting these neighborhood differences are explained by educational attainment. There was no evidence that the effect of the reminder intervention varied according to any of the other factors (see Supplementary Table 3 at www.jclinepi.com).

### 3.3 Impact on the nature of the response (consent rates)

Of the 806 who responded (by returning a completed consent form), 95.8% (772/806) consented to re-enroll into ALSPAC. Consent rates for ALSPAC's use of health and administrative records were: 92.4% (745/806) consent for health records, 93.4% (753/806) for school records, 93.1% (750/806) for further education records, 92.3% (744/806) for higher education records, 84.5% (681/806) for financial records, and 89.7% (723/806) for criminal conviction and caution records. We found no evidence (see Supplementary Table 4 at www.jclinepi.com) to suggest that any of the three interventions influenced whether a person consented to any of these (all *P*-values ≥0.2).

### 3.4 Harms

Our principle concern was differential consent outcomes between the trial groups—that is, that by running the trial we influenced participant consent decisions. As discussed above, we found no evidence to support this concern. No complaints were received about the trial, the materials, or that we were asking participants to consider this request.

### 3.5 Costings

The costings provided here are based on a production run of 10,000 to account for the economies of scale found in commercial printing. The costs (figures presented in GB£s at 2011 prices and adjusted for the changes in initial response rates found in this trial) are: the standard pack followed by the postcard reminder costs £22,010, the standard pack followed by the phone reminder costs £27,344, the designed pack followed by the postcard reminder costs £32,922, and the designed pack followed by the phone reminder costs £38,097. Assuming the observed 9.1% increase in response from the designed pack and phone reminder interventions, we would expect to receive 910 additional responses per 10,000 packs. This suggests there is an additional cost of £18 per extra responding participant as a result of adopting the effective intervention methods. The additional cost of the designed pack alone is £41 per extra respondent and the phone reminder alone costs £9 per extra respondent.

## 4 Discussion

The information pack and reminder interventions tested in this trial improved response when compared with standard approaches. There was some evidence (from planned subgroup analyses) that the phone reminder increased response rates from individuals with lower educational attainment, a demographic group who are historically less likely to participate in the study. Although these are clear advantages, the financial costs of the interventions are substantially higher than the standard approaches. We found no evidence that the three interventions tested had any influence on the enrollment and consent decisions of those who responded.

The evidence for which communication strategies optimize recruitment and retention rates, and at what cost, is limited. However, there are useful comparisons in studies evaluating interventions designed to improve trial recruitment rates [16], [27], [28] and study retention methods [15], [18], [29]. Previous studies evaluating the use of prior-notification mailings have found inconsistent effects: evidence suggests they increase questionnaire response [15], [29] but do not increase recruitment rates in clinical trials [16], [27], [28], [30]. Fox et al. [29], referring to the social exchange theories by Dillman [31], suggests that prior-notification mailings help establish trust in a study. In ALSPAC, the extent of participant trust is likely to be well established, potentially explaining why our prior-notification intervention had no measurable impact. Behavior theory suggests that the designed pack, which was personalized to the target audience, could reinforce the study's relationship with participants and thus increase response. This finding is consistent with previous studies [15], [29]. However, there is a wide range of approaches to improving information material design and some inconsistencies within the findings [12].

Previous studies have found that reminder follow-up is effective in increasing response [15] and that phone reminders have outperformed postal reminders [32], [33]. Our trial, based on a much larger sample size, provides new evidence to support this. Our fieldworkers recorded very few instances where participants asked questions about enrollment or the study's proposed use of their official records. This suggests that the value of the phone reminder may lie in maintaining and reinforcing our existing relationship with the participant rather than the call forming part of the informed consent process. Phone reminders made to parents were also effective, suggesting that studies can draw on the parents' commitment and loyalty to the study even after the child has reached adulthood. Although previous studies [18] have found that the effect of reminders increases in line with the number of reminders sent, we found evidence that the response after the postcard reminder was no higher than from those who were not contacted in the phone reminder group, suggesting there may be limited benefit in sending a second postal reminder. The cause of the interaction between the phone reminder and the standard pack design is not clear. It is also not clear why the phone reminder should have a greater benefit among individuals with low academic achievement, although, as discussed above, there is little evidence to suggest that the call was used as a means to supplement or question the information in the packs. Identifying this means of improving response rates from a group whose social factors are known to be predictive of nonresponse (i.e., those with low educational attainment) is a valuable finding, as reducing any selection bias introduced through selective participation is an important aim for cohort studies. These results will inform the rest of this wave of follow-up in ALSPAC, where we will drop the prior-notification postcard, retain the designed pack, and attempt to make phone reminder calls to all nonresponders. Importantly, a significant concern voiced [18] about this form of research—that the intervention methods may influence the consent decisions made by participants (i.e., the rates of consent or dissent to any given consent request)—was not observed. This implies that rigorous RCT evaluations of response strategies can be successfully embedded in cohort studies.

The trial's strengths are its rigorous methodology, a well-stratified sample with sufficient size to detect relatively small differences in response and the availability of sociodemographic information from the ALSPAC data set and existing linkage to education records. The analysis was hampered by small numbers in some sociodemographic groups (particularly nonwhite participants) and missing data when assessing sociodemographic characteristics. The RCT sample was not fully representative of the ALSPAC or PEARL sample, as it excluded individuals who had not participated for some time; therefore, the trial results may not be generalizable. These missing data should not affect the assessment of the interventions, as there are unlikely to be systematic differences between the intervention groups given the RCT design. As the reminder intervention took place only 3 weeks after the start of the trial, we were only able to examine the short-term effects of the prior-notification and information pack interventions. Because of the nature of the interventions, it was not possible for the individual to be “blind” to the intervention. This raises the possibility of “contamination” between intervention and control groups. We consider that there is limited risk of contamination as members of the same family (i.e., twins) received the same intervention and that the trial occurred after the individuals had finished compulsory education (and were therefore no longer clustered in schools).

This trial found evidence to suggest that certain modifications to the design and format of enrollment and consent materials may increase response rates and encourage response from participants with characteristics predictive of nonresponse. The response rate benefits we found need to be considered against the additional financial costs of these interventions. Studies should consider both these approaches when designing recruitment and consent campaigns, but we recommend that studies implement reminder phone calls and prioritize this intervention over the professional pack design where funding is limited. As most existing evidence considers adult participation in clinical RCTs, this trial adds new information as it evaluates differing communication approaches targeted at a sample of young adults, sampled from a cohort study, as they transition into adulthood. By successfully embedding this trial in a cohort study without adverse effects, we hope to encourage other studies to incorporate trials routinely to develop study methodologies. Although increased response rates have tangible benefits, perhaps this reassurance to experiment may have the greatest impact on the future success of cohort studies.

## Figures and Tables

**Table 1:** Response rates by intervention

Intervention	% of response rate (*n*/*n*)		% of difference in response	95% CI
Intervention group	Control group
1. Prior-notification intervention	10.9^a^ (107/979)	10.9^a^ (106/971)	0.01	−2.8, 2.8
2. Information pack intervention	12.3^a^ (120/978)	9.6^a^ (93/972)	2.7	−0.06, 5.5
3. Reminder intervention	23.4^b^ (165/704)	17.0^b^ (124/729)	6.4	2.3, 10.6
3a. Called the young person	33.0^c^ (60/182)	17.0 (124/729)	16.0	8.6, 23.3
3b. Called a parent	25.0^c^ (51/204)	17.0 (124/729)	8.0	1.5, 14.5
3c. SMS text or email sent	18.5^c^ (12/65)	17.0 (124/729)	1.5	−8.4, 11.3
3d. No contact	16.6^c^ (42/253)	17.0 (124/729)	−0.4	−5.7, 4.9

*Abbreviations*: CI, confidence interval; IIT, intention to treat; mIIT, modified ITT.

a ITT approach (Figure 1). Please note: denominators differ due to differences in the numbers allocated to each arm and differences in the numbers of individuals excluded from each arm once the trial had started.

b mITT approach (Figure 1).

c By necessity analysis conducted on a per-protocol basis.

**Table 2:** Interactions between the trial interventions

		Information pack intervention		*P*-value for interaction	Reminder intervention		*P*-value for interaction
Standard pack % response (*n*/*n*)	Designed pack % response (*n*/*n*)	Postcard reminder % response (*n*/*n*)	Phone reminder % response (*n*/*n*)
Prior-notification intervention	Sent	10.6^a^ (52/490)	11.3^a^ (55/489)	0.1	15.1^b^ (54/358)	23.0^b^ (82/356)	0.4
Not sent	8.5^a^ (41/482)	13.3^a^ (65/489)	18.9^b^ (70/371)	23.1^b^ (83/348)
Information pack intervention	Standard pack				15.3^b^ (57/372)	26.0^b^ (89/342)	0.05
Designed pack				18.8^b^ (67/357)	21.0^b^ (76/362)

*Abbreviations*: IIT, intention to treat; mIIT, modified ITT.

a ITT approach (Figure 1).

b mITT approach (Figure 1).
