# Supplementary material for: Professionally designed information materials and telephone reminders improved consent response rates: evidence from an RCT nested within a cohort study
Source: J Clin Epidemiol. 2015 Aug;68(8):877–87. doi: 10.1016/j.jclinepi.2015.03.014 (PMC4503222; doi:10.1016/j.jclinepi.2015.03.014)
Supplement: Supplementary Materials [file mmc1.pdf]

Information design and telephone reminders improved response rates in a cohort study: evidence from a randomised controlled trial.

Supplementary (Web Only) Materials.

Response by Socio-Demographic Characteristic

The response rates by category values were: sex (46.3% from females, 36.3% from males), ethnicity (39.8% from white participants, 34.6% from non-white participants), participation history (60.8% from those with a very high rate of historical participation, 25.3% from those with lower rates), educational attainment (46.3% from participants with five or more Grade A*-C KS4 results, 23.4% from those with less than five A*-C grades), low household income (33.0% from households eligible to receive FSM and 39.9% from households with income above the FSM threshold). It is important to note the relatively small sample sizes where the responding participant was from a low income household or was non-white.

Supplementary Figure 1: Response to the PEARL trial mailings, by time elapsed from the mailing of the information pack intervention.

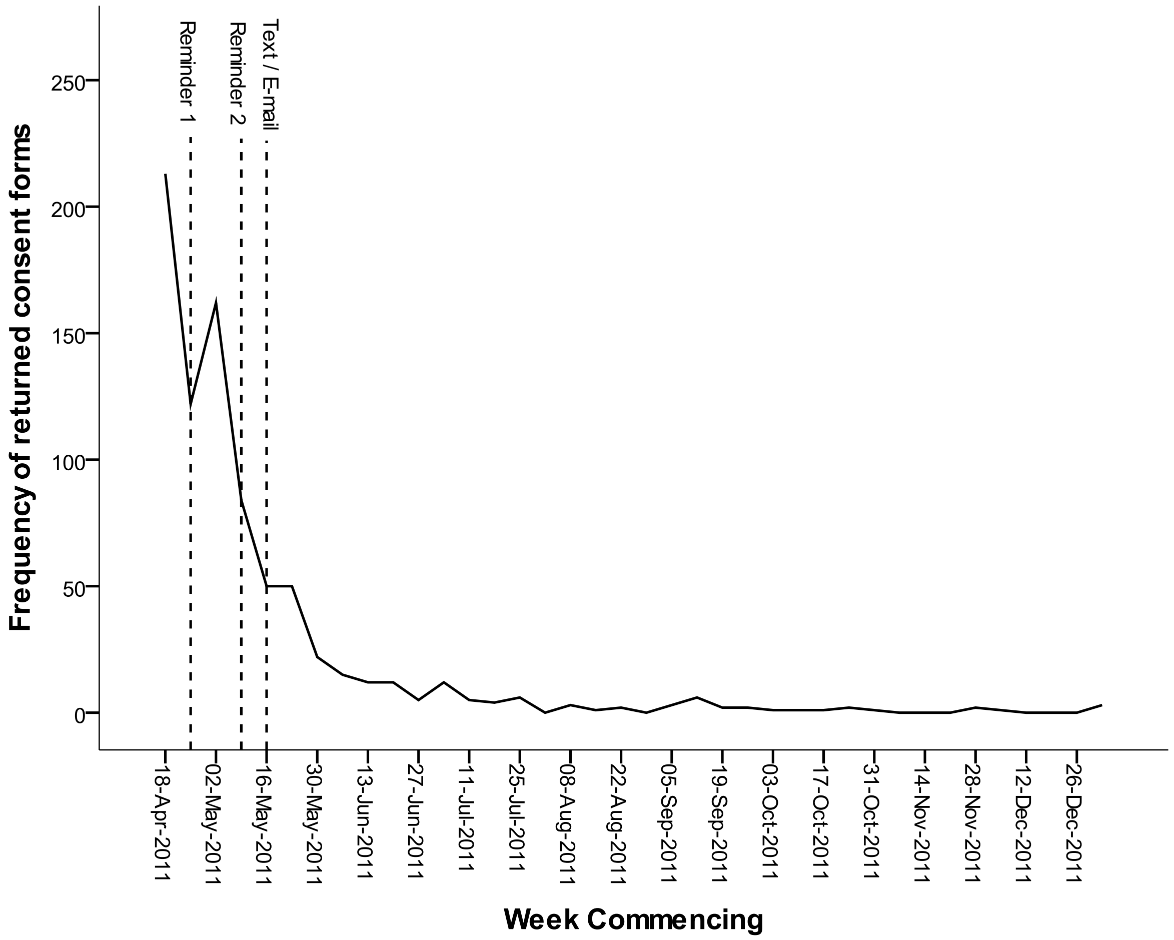

Supplementary Table 1. Response rates following the non-intervention reminder postcard, by prior-notification and information pack interventions.

Intervention	%Response Rate (n/n)		% Difference in Response	95% CI	P-value
	Intervention Group	Control Group			
1. Prior-notification intervention	18.1* (158/872)	16.9* (146/865)	1.2	-2.3, 4.8	0.5
2. Information Pack Intervention	16.2* (139/858)	18.8* (165/879)	-2.6	-6.1, 0.1	0.2

* ITT approach (Figure 1)

Supplementary Table 2: A comparison of socio-demographic characteristics between a national sample, the ALSPAC enrolled sample and the PEARL RCT sample and PEARL RCT responding sample

Characteristic	Category	National comparable sample[a]	ALSPAC enrolled sample[b]	PEARL RCT analysable sample	PEARL RCT responders
Child sex	Female	871,375/1,770,654 (49.2%)	5,470/11,008 (49.7%)	986/1,950[h] (50.6%)	456/806[m] (56.6%)
Child ethnicity	White	1,508,926/1,744,429 (86.5%)	10,505/10,933 (96.1%)	1,428/1,561[k] (91.5%)	568/614[n] (92.5%)
Participation History[c]	Very High	n/a	3,310/14,806 (22.4%)	880/1,950[h] (45.1%)	535/806[m] (66.4%)
Educational Attainment[e]	KS4 >= 5 grades A*-C	993,079/1,770,673 (56.1%)	6,493/10,805 (60.1%)	1,098/1,569[k] (70.0%)	508/618[n] (82.2%)
Low household income[f]	Eligible for FSM	218,033/1,745,353 (12.5%)	682/10,959 (6.2%)	91/1,561[k] (5.8%)	30/616[n] (4.9%)
Tertile of IMD[g]	1 (low)			527/1,950[h] (27.0%)	157/806[m] (19.5%)
	2	n/a	n/a	715/1,950[h] (36.7%)	330/806[m] (40.9%)
	3 (High)			708/1,950[h] (36.3%)	319/806[m] (39.6%)

[a] All pupils from the National Pupil Database (NPD) Key Stage 4 (KS4) English Government Maintained Establishments (GME) sample who are in the three academic year groups which include ALSPAC participants.

[b] All pupils, from English GMEs, who are from families that have enrolled in ALSPAC.

[c] 'Very High' indicates a participation history score of >=90.

[e] Academic attainment at the age of 16 years (achieving five or more assessments classed as grades A* to C, where A* signifies the highest level of attainment).

[f] Eligible for 'free school meals' (FSM), indicating a joint parental income of <=£16 000.

[g] Tertiles of IMD 2010, where 1 indicates the most deprived neighbourhoods (tertiles were set nationally and then mapped to ALSPAC participants using residential postcode)

[h] Denominator: the PEARL analysable sample

[k] Denominator: cases in the PEARL analysable sample, from English GMEs, with a valid record

[m] Denominator: PEARL RCT respondents

[n] Denominator: PEARL RCT respondents, from English GMEs, with a valid record

Supplementary Table 3: Sub-group analysis: response rates by intervention and socio-demographic characteristics.

		**Prior-notification Intervention***			**Information Pack Intervention***			**Reminder Intervention†**		
		% response			*% response*			*% response*		
**Characteristic**	**Category**	*Prior-Notification Postcard*	*No Prior-Notification Mailing*	*P‡*	*Designed Pack*	*Standard Pack*	*P‡*	*Phone Reminder*	*Postcard Reminder*	*P‡*
Sex	Male	8.5	8.3	0.9	9.77	7.04	0.7	21.3	13.6	0.3
	Female	13.3	13.4		14.69	12.07		25.7	20.8	
Ethnicity§	White	11.7	11.5	0.9	12.9	10.2	0.3	21.1	14.6	0.9
	Non-White	7.0	6.5		10.3	4.0		23.5	15.8	
Tertile of Indices Multiple Deprivation (IMD) 2007	Ttile 1 (low)	9.2	6.4	0.4	9.2	6.4	0.8	18.7	8.0	0.09
	Ttile 2	12.3	12.3		13.2	11.4		25.3	22.1	
	Ttile 3 (high)	10.9	12.9		13.6	10.2		25.7	19.3	

Free School Meals	Receives FSM	16.3	11.9	0.5	13.2	15.1	0.4	13.9	11.8	0.7
(FSM)	No FSM	11.0	11.2		12.8	9.4		22.0	15.1	
# Key Stage 4 Grades	Less than Five	6.8	6.3	0.9	6.8	6.4	0.5	16.0	4.3	0.004
A*-C	Greater or Equal to Five	13.3	13.1		15.3	11.1		24.2	20.7	
ALSPAC Participation	<90	6.5	6.2	0.7	6.4	6.3	0.2	15.7	9.0	0.2
History Score	>=90	16.2	16.7		19.3	13.6		37.5	30.6	

[*] ITT approach(Figure 1)

[†] mITT approach (Figure 2)

[‡] Test for interaction

[§] The PEARL RCT sample has relatively small numbers of non-white participants (total n=135)

Supplementary Table 4: Consent rates by trial intervention and consent category

**Consent Category**	**Prior-notification Intervention*** *% consented (n/n)*			**Information Pack Intervention*** *% consented (n/n)*			**Reminder Intervention†** *% consented (n/n)*		
	Prior-notification Postcard	*No Prior-Notification Mailing*	*P‡*	*Designed Pack*	*Standard Pack*	*P‡*	*Phone Reminder*	*Postcard Reminder*	*P‡*
Participation in ALSPAC	95.3 (102/107)	98.1 (104/106)	0.6	96.7 (116/120)	96.8 (90/93)	0.6	91.5 (151/165)	96.0 (119/124)	0.3
Linkage to Health Records	92.5 (99/107)	97.2 (103/106)	0.2	96.7 (116/120)	92.5 (86/93)	0.2	87.9 (145/165)	90.3 (112/124)	0.6
Linkage to School Records	93.5 (100/107)	98.1 (104/106)	0.2	96.7 (116/120)	94.6 (88/93)	0.5	87.9 (145/165)	91.9 (114/124)	0.3
Linkage to Further Education Records	92.5 (99/107)	97.2 (103/106)	0.2	95.8 (115/120)	93.6 (87/93)	0.5	87.9 (145/165)	91.9 (114/124)	0.3

Linkage to Higher Education Records	95.5 (99/107)	96.2 (102/106)	0.2	94.2 (113/120)	94.6 (88/93)	1.0	86.7 (143/165)	91.9 (114/124)	0.3
Linkage to Financial and Benefit Records	86.0 (92/107)	90.6 (96/106)	0.1	89.2 (107/120)	87.1 (81/93)	0.8	80.0 (132/165)	80.7 (100/124)	1.0
Linkage to Criminal Records	93.5 (100/107)	94.3 (100/106)	0.4	94.2 (113/120)	93.6 (87/93)	1.0	81.8 (135/165)	88.7 (110/124)	0.2

[*] ITT approach (see Figure 1 in manuscript)

[†] mITT approach (see Figure 1 in manuscript)

[‡] Fishers exact test
